# Medical Coroner

**Published:** 1842-10-01

**Authors:** 


### MEDICAL CORONER.

At the ensuing annual election in Philadelphia, a Coroner is to be chosen for three years. As one of the candidates for this situation is a physician, we take the occasion to rëiterate the opinion heretofore expressed in this Journal, that the duties of the office demand, for their adequate discharge, a knowledge of anatomy and medical jurisprudence, which cannot be found out of the medical profession. The propriety of selecting a physician for this post has been very generally recognized. The present Coroner of Middlesex, Mr. Wakley, was elected some years ago, by a very large majority, on the ground of his peculiar professional qualifications, and the practice prevails, likewise, in New York of selecting a physician for the office. If it be given to a non-medical man, he is obliged to discharge his most responsible duties second hand, or neglect them, as has been generally the case in Philadelphia.

We noticed with pleasure that the names of several most respectable physicians were recently submitted to the two political parties, for the office of Coroner. We must express our regret that the nominating delegates of one party passed over a physician, entirely qualified for the post by professional knowledge, intelligence, and integrity ; and as there is but one medical man now in the field, we feel bound to ask for him a general support, without reference to party distinction.

